# The first report of *Aelurostrongylus falciformis *in Norwegian badgers (*Meles meles*)

**DOI:** 10.1186/1751-0147-48-6

**Published:** 2006-06-13

**Authors:** Rebecca K Davidson, Kjell Handeland, Bjørn Gjerde

**Affiliations:** 1 Section for Wildlife Diseases, National Veterinary Institute, P.O. Box 8156 Dep., NO-0033 Oslo, Norway; 2 Parasitology Laboratory, Section for Microbiology, Immunology and Parasitology, Institute for Food Safety and Infection Biology, Norwegian School of Veterinary Science, P.O. Box 8146 Dep., NO-0033 Oslo, Norway

## Abstract

The first report of *Aelurostrongylus falciformis *(*Schlegel 1933*) in Fennoscandian badgers is described. Routine parasitological examination of nine Norwegian badgers, at the National Veterinary Institute during 2004 and 2005, identified *A*. *falciformis *in the terminal airways of five of the animals. The first stage larvae (L1) closely resembled, in size and morphology, those of *Angiostrongylus vasorum *(*Baillet 1866*). The diagnosis for both *A*.* falciformis *and *A*. *vasorum *is frequently based on the identification of L1 in faeces or sputum. The potential for misclassification of an *A*. *falciformis *infection as *A*.* vasorum*, where larval identification is the only diagnostic method used, is discussed.

## Background

*Aelurostrongylus falciformis *(*Schlegel 1933*) is a metastrongyle lung nematode of European badgers (*Meles meles*) and has been reported in continental Europe [1,2] and Great Britain [3] but not Fennoscandia. Other lung nematodes seen in European badgers include the metastrongyles *Angiostrongylus vasorum *(*Baillet 1866*) [4,5], *Crenosoma *sp. (*Molin 1861*) [1-4], *Aelurostrongylus pridhami *(*Anderson 1962*) [5], as well as the trichuroid nematode *Capillaria aerophila *(*Creplin 1839*) [2]. *A*.* vasorum *is considered to be absent from the Scandinavian Peninsula. However, recently it has been found on the island of Sydkoster off the south west coast of Sweden [6] close to the Norwegian border. This parasite has its predilection site in the pulmonary artery and right ventricle of the heart. The diagnosis however, as for *A*. *falciformis*, is frequently based on the identification of first stage larvae (L1) in faeces or sputum [7]. *Crenosoma *sp. and *Capillaria aerophila *infections can be differentiated from those of *A*. *vasorum *and *A*. *falciformis *on the basis of their typical L1 (*Crenosoma *sp.) and eggs (*Capillaria aerophila*).

Nine badgers from the Oslo and Akershus regions were sent to the National Veterinary Institute, Oslo, during 2004 and 2005 (Table 1). Routine post-mortem was carried out and revealed trauma as the cause of death in seven of the animals whereas two had been shot for humane reasons. Parasitological examination of the respiratory tract, cardiovascular system and faeces was also carried out.

**Table 1:** Information regarding the nine badgers (*Meles meles*) submitted to the National Veterinary Institute, Oslo during the course of 2004 and 2005 for post-mortem examination and examined for lung worms.

**Badger ID**	**Sex**	**Age**	**Cause of death**	**Municipality of origin**	***Aelurostrongylus falciformis*(lpg)**	***Crenosoma melesi*(lpg)**
1	female	adult	trauma	Vestby	positive (7)	positive (0.3)
2	female	adult	trauma	Oslo	negative	negative
3	male	adult	trauma	Vestby	negative	positive^**a**^
4	female	adult	trauma	Oslo	negative	negative
5	male	adult	trauma	Vestby	positive (49)	positive (11)
6	unknown	adult	trauma	Vestby	negative	negative
7	female	adult	shot	Grue	positive (460)	negative
8	male	juvenile	shot	Vestby	positive^**a**^	negative
9	male	adult	trauma	Vestby	positive^**a**^	negative

^**a **^Baermannisation of the faeces not carried out, diagnosis based on the presence of larvae in bronchial scrapes.

The trachea and bronchi were dissected; two scrapes from the mucous membrane were taken and examined for nematode eggs and larvae. Biopsies, from each lung lobe, were fixed in 10% formalin and embedded in paraffin. They were then cut at 5μm and stained with haematoxylin and eosin for histological examination. The right side of the heart was incised and this incision extended along the pulmonary artery to look for adult *A*.* vasorum*. At least two 10 gram faecal samples per animal, from some of the badgers, were set up in Baermann apparatus for 6 hours and then examined for faecal larval burden, larvae per gram (lpg); the larvae collected were stored in 70% ethanol. Larvae from the respiratory tract and faeces were examined under magnification, those with a wavy tail were digitally photographed: length and width measurements were taken (Leica Image Manager IM50 Measurement Module, Version 4.0, Release 106). The width was measured at the widest point.

Nematode larvae with a curvy tail and a notch, suggestive of *A*. *vasorum *infection, were found for the first time in bronchial scrapes. A more advanced examination of the lungs in badger 1 was carried out. The lungs were dissected to bronchiolar level and any adult nematodes were carefully removed and placed in 70% ethanol. Larvae were extracted from the uterus of an adult female and compared to those found in the respiratory tract and faeces. The adult nematodes and larvae were examined under magnification, digitally photographed and measured. All adult nematodes fragmented during extraction, and their overall length could not be measured. Width measurements were taken just anterior to the spicules in the males and level with the uterus in the females. The length of the male spicules and accessory spicules were recorded.

**Figure 1:**
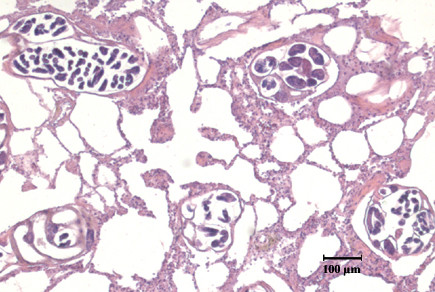
Cross-sections of adult *Aelurostrongylus falciformis *in the terminal airways of a Norwegian badger (Leica DC300 Digital Camera). Bar = 100μm.

**Figure 2:**
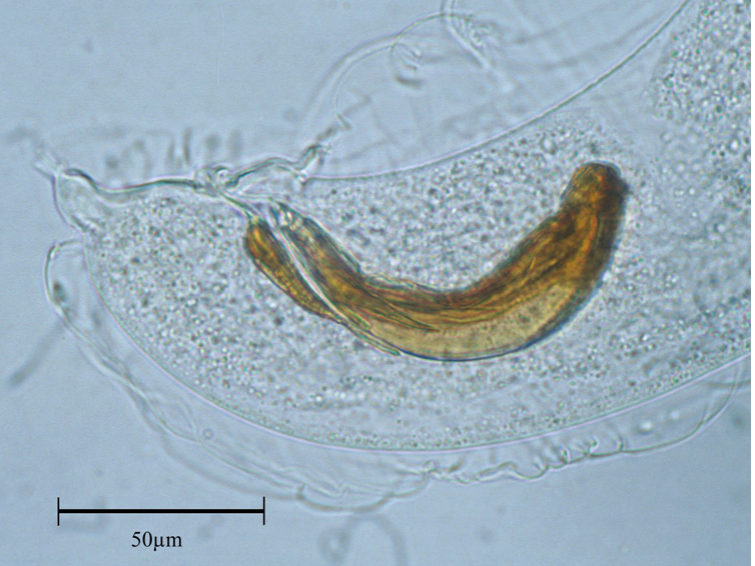
Posterior end of a male *Aelurostrongylus falciformis *found in the lungs of a Norwegian badger. Evident are the spicules, the accessory chinitous piece and the small bursa (Leica MPS 60 Camera). Bar = 50μm.

*A*. *falciformis *was found in five badgers and three had *Crenosoma melesi *infections. Two of the badgers were simultaneously infected with both parasites. Histological examination confirmed the location of *A*. *falciformis *within the terminal airways (Figure 1). Width measurements of adult male and female nematodes, as well as the length of the male spicules (Figure 2) and accessory spicule are given in Table 2, which also shows reference values given in the literature for *A*. *falciformis *and *A*. *vasorum*. No *A*. *vasorum *was found during the dissection of the heart and pulmonary artery.

**Table 2:** Several dimensions of adults and larvae of *Aelurostrongylus falciformis *as found in Norwegian badgers (with the median value given in brackets), as well as analogous dimensions of *A. falciformis *and *Angiostrongylus vasorum *according to reference literature.

**Dimensions**	***Aelurostrongylus falciformis – Norwegian badgers***	***Aelurostrongylus falciformis ***[8;9;12]	***Angiostrongylus vasorum ***[7;13]
Male width (μm)	48 – 81.5 (69)	50	170 – 235
Female width (μm)	84.5 – 148.5 (105)	60 – 172	220 – 306
length (μm)	95 – 133 (100)	80	400 – 500
Accessory spicule length (μm)	39 – 52.5 (46)	40	Not applicable
L1 larval length (μm)	213 – 353 (248)	220 – 370	310 – 399
L1 larval width (μm)	9 – 19 (14)	15 – 17	13 – 17

**Figure 3:**
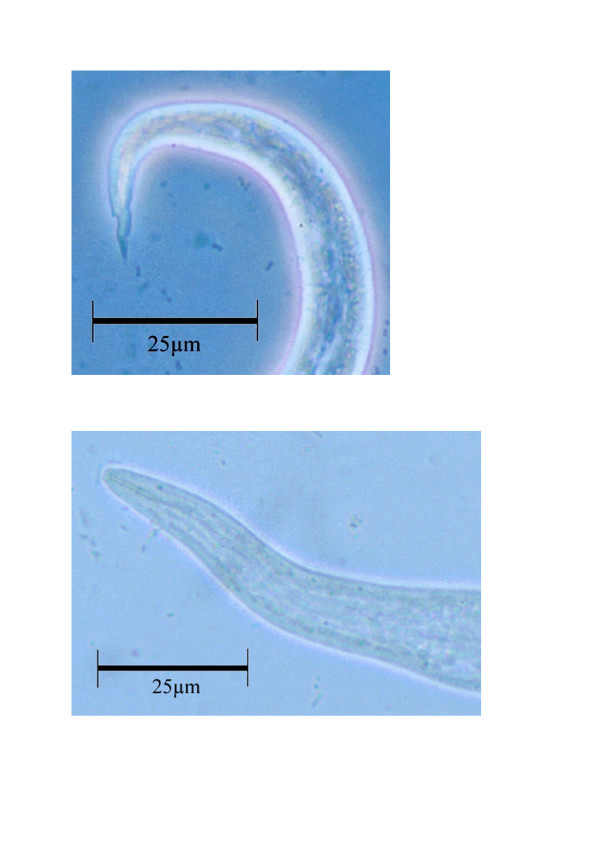
The tail (**a**) and anterior end (**b**) of the L1 larvae found in a Norwegian badger infected with *Aelurostrongylus falciformis*. (Leica MPS 60 Camera; **a **– phase contrast; **b **– bright field). Bar = 25μm.

Larval burden varied considerably between the positive animals (Table 1). Morphologically, the larvae appeared identical to those obtained from an adult female. The length and width of the isolated L1 were within the reference range given for *A*.* falciformis *(Table 2). The larvae had a wavy tail with a notch (Figure 3a) and the anterior end of the larvae had what could be interpreted as a cephalic button (Figure 3b) resembling the L1 of *A*. *vasorum *[6,7]. The parasite was identified as *A*. *falciformis *given the location of the adult nematodes in the lung tissue, the length of the male spicules, the presence of an accessory spicule, and the size and morphology of the larvae [8,9].

The present study is believed to be the first report of *A*.* falciformis *infection in Fenno-Scandinavian badgers. The L1 length was within the published reference range for *A*. *falciformis*, which overlaps the published L1 range for *A*.* vasorum*. *A*. *vasorum *can be found in ectopic locations [10], but the male nematodes, from the reported badger's lungs, had short spicules and an accessory chitinous piece, excluding *A*.* vasorum *from the differential list. Nevertheless, the great similarity between the L1 stages of *A*.* falciformis *and *A*. *vasorum*, in size and general morphology, needs to be considered.

The prevalence of *A*. *falciformis *found in European badgers, as well as the diagnostic criteria underlying the diagnosis, varies considerably between studies and needs further investigation. Magi et al. (1999) [1] identified adult *A*. *falciformis *and L1 in the lungs of 10 of 19 (53%) Italian badgers. In a British study of 118 badgers for tuberculosis, *A*. *falciformis *was recorded incidentally in one animal [3]. In Spain, Millán et al. (2004) [4] reported *A*.* vasorum *larvae in the faeces of 24 of 26 (92%) badgers; however, they were unable to find adult nematodes in the pulmonary artery or the right ventricle of the heart. Another Spanish badger study [5] reported the presence of both *A*. *vasorum *and *A*. *pridhami*, the latter is a parasite of wild mink in North America [11], without describing the identification methods used. It may be possible that an infection with *A*. *falciformis*, particularly in areas where *A*.* vasorum *is endemic, could be misinterpreted as *A*.* vasorum*, when larval identification is the only diagnostic method used.
